# The associations between psychological distress and healthcare use in patients with non-cardiac chest pain: does a history of cardiac disease matter?

**DOI:** 10.1186/s12888-018-1689-8

**Published:** 2018-06-05

**Authors:** Ghassan Mourad, Tiny Jaarsma, Anna Strömberg, Erland Svensson, Peter Johansson

**Affiliations:** 1 Department of Social and Welfare Studies, Linköping University, Kungsgatan 40, S-601 74 Norrköping, Sweden; 2 Department of Medical and Health Sciences, Linköping University, Linköping, Sweden; 3 Department of Cardiology and Department of Medical and Health Sciences, Linköping University, Linköping, Sweden; 4 Formerly Swedish Defence Research Agency, Stockholm, Sweden; 5 Department of Internal Medicine and Department of Medical and Health Sciences, Linköping University, Norrköping, Sweden

**Keywords:** Cardiac anxiety, Cardiac disease, Depressive symptoms, Fear of body sensations, Healthcare visits, Non-cardiac chest pain, Somatization

## Abstract

**Background:**

Psychological distress such as somatization, fear of body sensations, cardiac anxiety and depressive symptoms is common among patients with non-cardiac chest pain, and this may lead to increased healthcare use. However, the relationships between the psychological distress variables and healthcare use, and the differences in relation to history of cardiac disease in these patients has not been studied earlier. Therefore, our aim was to explore and model the associations between different variables of psychological distress (i.e. somatization, fear of body sensations, cardiac anxiety, and depressive symptoms) and healthcare use in patients with non-cardiac chest pain in relation to history of cardiac disease.

**Methods:**

In total, 552 patients with non-cardiac chest pain (mean age 64 years, 51% women) responded to the Patient Health Questionnaire-15, Body Sensations Questionnaire, Cardiac Anxiety Questionnaire, Patient Health Questionnaire-9 and one question regarding number of healthcare visits. The relationships between the psychological distress variables and healthcare visits were analysed using Structural Equation Modeling in two models representing patients with or without history of cardiac disease.

**Results:**

A total of 34% of the patients had previous cardiac disease. These patients were older, more males, and reported more comorbidities, psychological distress and healthcare visits. In both models, no direct association between depressive symptoms and healthcare use was found. However, depressive symptoms had an indirect effect on healthcare use, which was mediated by somatization, fear of body sensations, and cardiac anxiety, and this effect was significantly stronger in patients with history of cardiac disease. Additionally, all the direct and indirect effects between depressive symptoms, somatization, fear of body sensations, cardiac anxiety, and healthcare use were significantly stronger in patients with history of cardiac disease.

**Conclusions:**

In patients with non-cardiac chest pain, in particular those with history of cardiac disease, psychological mechanisms play an important role for seeking healthcare. Development of interventions targeting psychological distress in these patients is warranted. Furthermore, there is also a need of more research to clarify as to whether such interventions should be tailored with regard to history of cardiac disease or not.

## Key points

- Cardiac anxiety was the main associate to healthcare use
- Depressive symptoms were only indirectly associated with healthcare use
- The direct and indirect effects between psychological distress and healthcare use were stronger in patients with a history of cardiac disease

## Background

Non-Cardiac Chest Pain (NCCP) is a common condition [1] with substantial impact on patients’ psychological wellbeing, health-related quality of life, and healthcare use [2–4]. Many patients with NCCP are not convinced by their ‘ruled out’ cardiac diagnosis, and lack an explanation for their chest pain [5–7]. They continue to experience chest pain and avoid activities that they think might be harmful to their heart [1, 8–10], leading to substantial use of healthcare and societal resources [11].

**Fig. 1:**
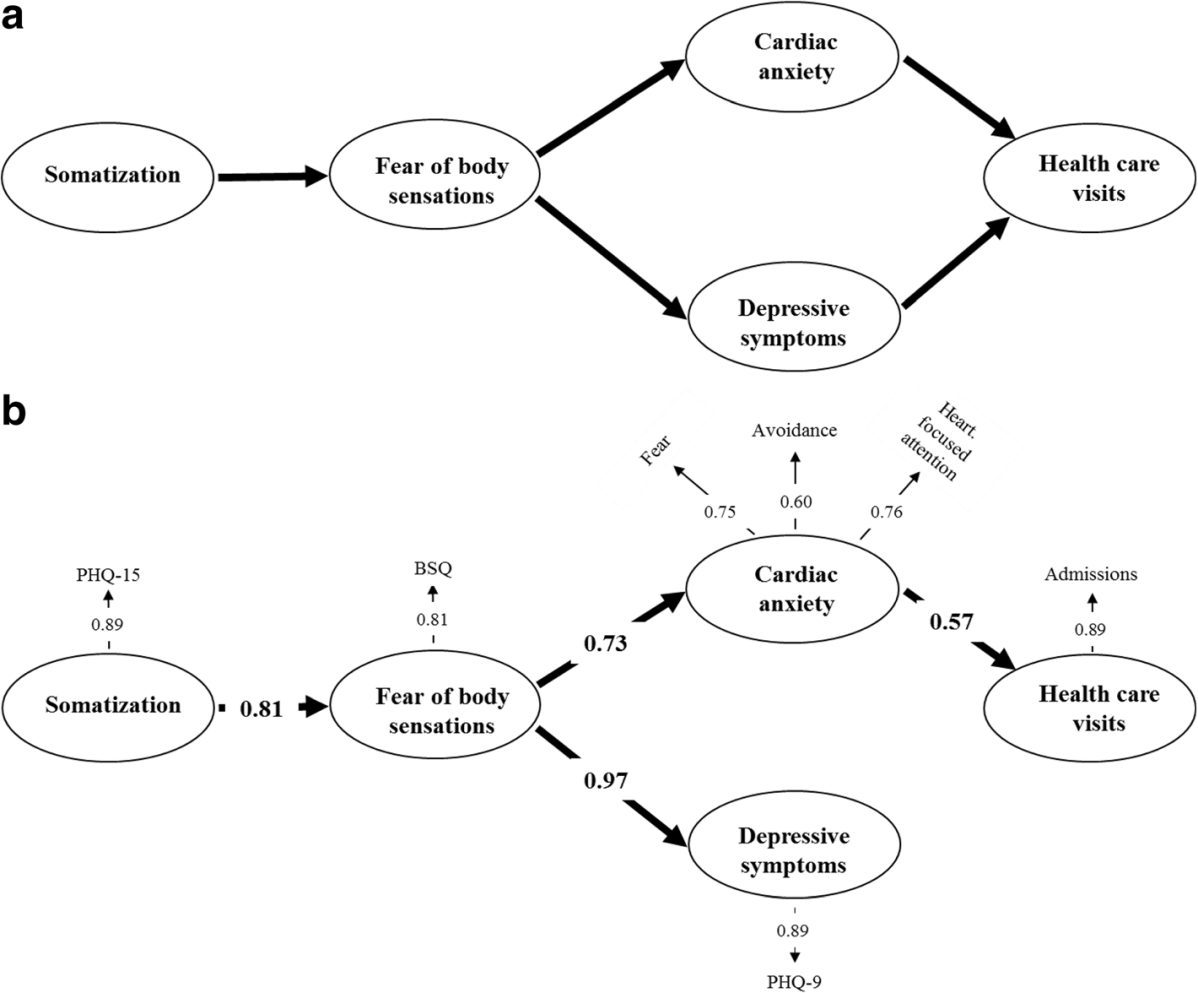
**a** + **b** The pre-assumed model of the associations between somatization, fear of body sensations, cardiac anxiety, depressive symptoms, and healthcare use in patients with non-cardiac chest pain. Chi-Square = 75.94, df = 10, *P*-value = 0.00000, RMSEA = 0.110, CFI = 0.95. *BSQ* Body Sensations Questionnaire, *CAQ* Cardiac Anxiety Questionnaire, *PHQ-9* Patient Health Questionnaire-9, *PHQ-15* Patient Health Questionnaire-15

Different psychological problems such as fear of body sensations, cardiac anxiety, and depressive symptoms may be involved in the process of symptom interpretation and healthcare use [12, 13]. According to the “Fear-Avoidance Model”, the reaction to the experience of severe pain is primarily characterized by fear, which is followed by either confrontation or avoidance. While confrontation reduces the fear, avoidance leads to maintenance and exacerbation of the fear [14, 15]. Informed by the Fear-Avoidance model, a model was suggested implying that patients who experience recurrent and persistent chest pain that they perceive as threatening may express psychological distress in physical symptoms (i.e. somatization) and experience pain-related fear, which in turn can lead to cardiac anxiety and depressive symptoms, and thus increase healthcare use due to fear of having a cardiac event (Fig. 1a).

However, this model has not been evaluated in patients with NCCP. Furthermore, patients with history of Cardiac Disease (CD) (i.e. angina pectoris, myocardial infarction, and/or heart failure) can also have NCCP [16, 17], but few studies distinguish between these groups with regard to psychological presentation and healthcare use [16–19]. Since patients who have been diagnosed with CD are informed by healthcare providers that they should pay attention to and seek immediate care in case of persistent chest pain [20, 21], these patients may consider chest pain as more fearful and threatening than patients with no history of CD. Therefore, our aim was to explore and model the associations between different variables of psychological distress (i.e. somatization, fear of body sensations, cardiac anxiety, and depressive symptoms) and healthcare use in patients with NCCP in relation to history of CD.

## Methods

This is a secondary analysis of a cross sectional study [13]. In the original analysis, the prevalence of fear of body sensations, cardiac anxiety, depressive symptoms and their relations to number of healthcare visits was studied. In this analysis, Structural Equation Modeling (SEM) analyses were used to explore and model the associations between somatization, fear of body sensations, cardiac anxiety, depressive symptoms and healthcare visits in patients with NCCP, divided into two groups based on experience of CD.

### Study participants and procedures

The recruitment process and first results are described in the earlier paper [13]. In brief, 2271 patients older than 18 years who had sought medical care due to chest pain and been diagnosed with NCCP after ruling out acute myocardial infarction between October 2013 and January 2014 were approached within 1 month from the day of discharge from four hospital in southeast Sweden. NCCP was determined using the International Classification of Diseases (ICD) 10-codes: R07.2, R07.3, R07.4, and Z03.4.

The study information, an informed consent form, and the questionnaires were sent by post to all eligible patients together with a pre-stamped envelope for response. Patients had the possibility to contact the research team in case of questions. For study participation, patients were asked to sign and return the informed consent form together with the completed questionnaires. In total, 552 patients were included in the study.

### Data collection and measurements

All data including socio-demographics, health complaints and history of CD was self-reported. Heart failure was included as part of the CD since the diagnosis is cardiac and is often associated with psychological distress. Self-reported data by patients on e.g. chronic diseases is reported to be fairly accurate compared to physician reports [22].

#### Somatization

The Patient Health Questionnaire-15 (PHQ-15) was used to measure somatization, which is report of somatic symptoms that have no pathophysiological cause [23, 24]. The PHQ-15 comprises 15 items/physical symptoms with scores between 0 and 30. Scores of 10 or higher indicate at least medium severity. The PHQ-15 has high reliability and validity [23]. In the present study, the Cronbach’s α coefficient was 0.85.

#### Fear of body sensations

Fear of body sensations, such as palpitations, dizziness and sweating, was measured with the Body Sensations Questionnaire (BSQ) containing 17 items and scores between 17 and 85. Higher scores indicate more fear of body sensations [25]. The BSQ is reliable and valid [10, 13, 25]. In the present study, the Cronbach’s α coefficient was 0.93.

#### Cardiac anxiety

Cardiac anxiety was measured using the Cardiac Anxiety Questionnaire (CAQ). The questionnaire comprises 18 items and the scores range between 0 and 72, and higher scores indicate greater cardiac anxiety. The CAQ can be divided into three subscales (i.e. fear, avoidance, and heart-focused attention). The CAQ has demonstrated good reliability and validity [26]. In the present study, the Cronbach’s α coefficient was 0.90 for the total CAQ and 0.84, 0.89 and 0.77 for the subscales fear, avoidance, and heart-focused attention.

#### Depressive symptoms

The Patient Health Questionnaire-9 (PHQ-9) was used to measure depressive symptoms. The PHQ-9 comprises 9 items and scores between 0 and 27. At a score of 10 or higher, the PHQ-9 has a sensitivity for major depression of 88%, a specificity of 88%, and a positive likelihood ratio of 7.1. The PHQ-9 has shown to have high internal consistency [27]. In the present study, the Cronbach’s α coefficient was 0.87.

#### Healthcare visits

The number of healthcare visits was determined by asking the participants the following self-developed question: “*In the last year, how many times did you seek care due to chest pain?”* Answers were predetermined to the categories: “1, 2-3, or >3”.

### Analytic strategy and statistical analysis

Descriptive statistics, comparative analyses, and SEM were used to analyse the data. To compare background characteristics of the participants with or without history of CD, categorical variables were tested with Chi-Square tests while continuous variables were analysed with Student’s t-test or Mann-Whitney U-test, depending on distribution of normality. SEM was used to explore and model the associations between the different variables of psychological distress and healthcare visits in patients with NCCP with or without history of CD. To calculate the differences between the associations in the SEM models of those with or without history of CD, Fisher r to z transformation was used. Analyses of potential non-linear relationships between somatization, fear of body sensations, cardiac anxiety, and depressive symptoms were performed by means of Distance-Weighted Least Squares regression (DWLS).

SEM allows modeling of the relationships between variables that may have inter-dependent associations. Associations within the model were analysed using maximum likelihood and are described using standardized coefficients. Goodness of fit of the model was analysed with Chi-Square, the Root Mean Square Error of Approximation (RMSEA), and the Comparative Fit Index (CFI). These estimates provide information on how well the model represent our population of patients with NCCP with or without CD. A non-significant Chi-Square value, a RMSEA < 0.06 and a CFI ≥ 0.95 indicate a good model fit [28].

SPSS version 23.0 was used to perform the descriptive and comparative analyses, and LISREL 8.30 software [28] was used to perform the SEM analyses. Significance levels were set at *p* < 0.05 for SPSS analyses and t-value of ≥1.96 for LISREL analyses regarding factor loadings and effects. Curve-linearity analyses between the latent variables were performed by SYSTAT [29].

## Results

### Study participants

Of the 2271 patients who were approached, 680 (30%) agreed to participate, but only 552 (24%) fulfilled the criteria and were included in the study. Study participants had a mean age of 64 (±17) years and 51% were women, 67% were married/cohabiting and 55% were retired (Table 1). Non-respondents were significantly younger (54 ± 20 years, *p* < 0.001) and those who declined participation were significantly older (70 ± 17 years, *p* < 0.001) compared to study participants.

**Table 1:** Characteristics of patients with non-cardiac chest pain

	Total^a^ (*N* = 552)	History of cardiac disease (*n* = 188)	No history of cardiac disease (*n* = 360)	*p*-value
Sex n (%)				< 0.001
Males	271 (49)	112 (60)	157 (44)	
Females	281 (51)	76 (40)	203 (56)	
Age year (mean ± SD)	64 ± 17	71 ± 13	60 ± 17	< 0.001
Married/cohabiting n (%)	370 (67)	117 (62)	251 (70)	0.062
Educational level n (%)				< 0.001
Compulsory school	185 (34)	91 (48)	91 (25)	
High school	216 (39)	60 (32)	156 (43)	
University	150 (27)	37 (20)	112 (31)	
Work status n (%)				< 0.001
Working	152 (28)	27 (14)	125 (35)	
Retired	302 (55)	132 (70)	167 (46)	
Sick-leave/disability pension	40 (7)	16 (9)	24 (7)	
Other	57 (10)	13 (7)	44 (12)	
Smoking n (%)				0.471
None/Previous smokers	493 (89)	166 (88)	325 (90)	
Smokers	59 (11)	22 (12)	35 (10)	
Healthcare contacts n (%)				< 0.001
≤ 1 contacts per year	331 (60)	88 (47)	241 (67)	
2-3 contacts per year	145 (26)	55 (29)	88 (24)	
> 3 contacts per year	76 (14)	45 (24)	31 (9)	
History of illness n (%)				
Angina Pectoris	114 (21)	114 (61)	0 (0)	–
Asthma/bronchitis	66 (12)	22 (12)	43 (12)	0.725
Chronic obstructive pulmonary disease	37 (7)	24 (13)	13 (4)	< 0.001
Diabetes	64 (11)	45 (24)	19 (5)	< 0.001
Gastric ulcer	48 (9)	18 (10)	30 (8)	0.213
Heart failure	55 (10)	55 (29)	0 (0)	–
Hypertension	247 (45)	116 (62)	131 (36)	< 0.001
Mental disorder	107 (20)	39 (21)	67 (19)	0.739
Musculoskeletal pain	307 (55)	104 (55)	202 (56)	0.087
Myocardial infarction	104 (19)	104 (55)	0 (0)	–
Reflux/heartburn	234 (43)	67 (36)	166 (46)	0.005
Number of conditions (mean ± SD)	4 ± 2	5 ± 2	3 ± 2	< 0.001

^a^data regarding history of cardiac disease missing for four patients

About 34% of the participants had previous diagnosis of CD (i.e. angina pectoris, myocardial infarction, and/or heart failure). These participants were significantly older (71 vs. 60 years), more males (60 vs. 44%), had lower educational levels, reported more conditions (5 vs. 3) and had larger number of healthcare visits compared to patients with no history of CD. Furthermore, patients with history of CD reported significantly higher scores in all psychological distress variables except for fear of body sensations (Table 2).

**Table 2:** Psychological distress in patients with non-cardiac chest pain, (mean ± SD)

	Total (*N* = 552)	History of cardiac disease (*n* = 188)	No history of cardiac disease (*n* = 360)	*p*-value
Somatization				
PHQ-15 total score	10.0 ± 5.4	10.8 ± 5.6	9.5 ± 5.2	0.007
Fear of body sensations				
BSQ total score	31.4 ± 12.1	31.5 ± 12.9	31.3 ± 11.7	0.837
Cardiac anxiety				
CAQ total score	24.6 ± 13.0	28.2 ± 14.5	22.7 ± 11.8	< 0.001
- Fear mean score	1.6 ± 0.9	1.8 ± 0.9	1.6 ± 0.8	0.010
- Avoidance mean score	1.3 ± 1.0	1.7 ± 1.1	1.1 ± 0.9	< 0.001
- Heart-focused attention mean score	1.1 ± 0.8	1.2 ± 0.9	1.0 ± 0.7	0.017
Depressive symptoms				
PHQ-9 total score	6.4 ± 5.9	7.4 ± 6.6	5.8 ± 5.4	0.002

*BSQ* Body Sensations Questionnaire, *CAQ* Cardiac Anxiety Questionnaire, *PHQ-9* Patient Health Questionnaire-9, *PHQ-15* Patient Health Questionnaire-15

### Modeling the associations between somatization, fear of body sensations, cardiac anxiety, depressive symptoms and healthcare use

**Fig. 2:**
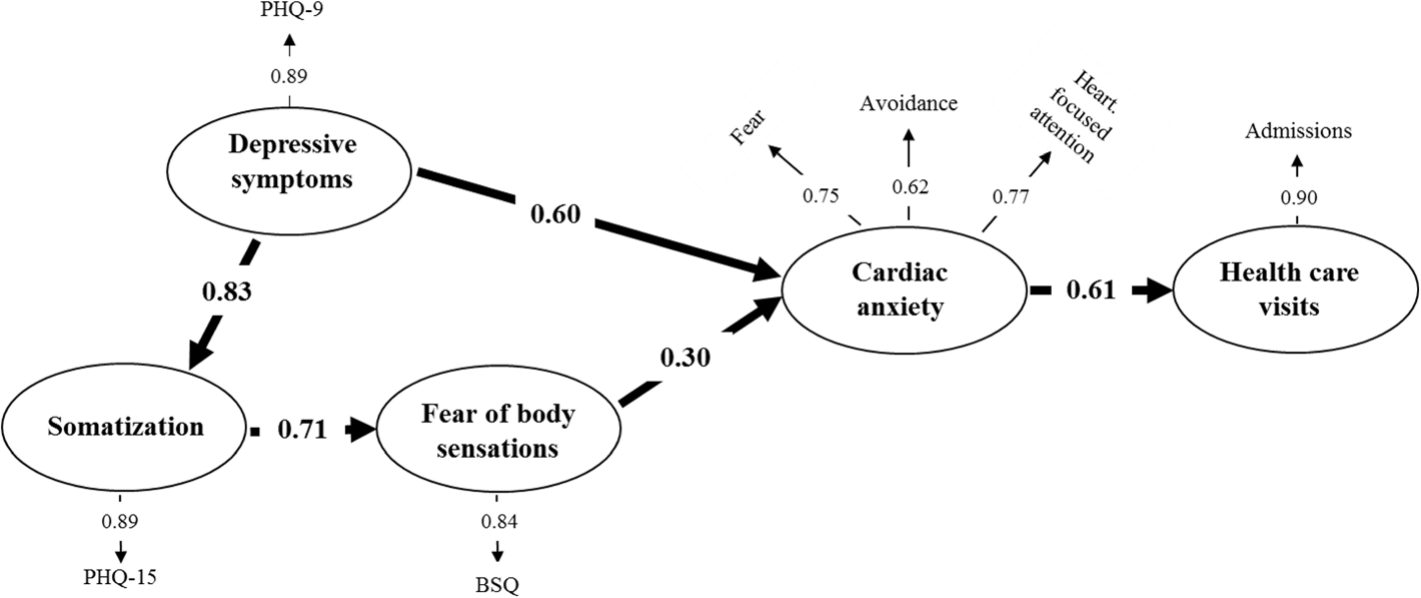
The revised model of the associations between somatization, fear of body sensations, cardiac anxiety, depressive symptoms, and healthcare use in patients with non-cardiac chest pain. Chi-Square = 1.93, df = 5, P-value = 0.85819, RMSEA = 0.000, CFI = 0.99. *BSQ* Body Sensations Questionnaire, *CAQ* Cardiac Anxiety Questionnaire, *PHQ-9* Patient Health Questionnaire-9, *PHQ-15* Patient Health Questionnaire-15

The SEM analysis of the assumed paths (Fig. 1a) between somatization, fear of body sensation, cardiac anxiety, depressive symptoms and healthcare visits in patients with NCCP showed a poor fit (Chi-Square statistics 75.94, df = 10, *p* < 0.001: RMSEA = 0.110: and CFI = 0.95) (Fig. 1b). The model showed that there was no direct association between depressive symptoms and healthcare visits. Since a 2015 paper exploring fear avoidance in pain patients and using SEM analysis, reported that depressive symptoms could be seen as a preconditioning variable for fear avoidance and functioning [30], our model was revised to explore if depressive symptoms would occur more downstream in the model. Thus, by the possible associations of depressive symptoms with somatization, and/or fear of body sensations, and/or cardiac anxiety, depressive symptoms could have an indirect effect on healthcare visits. In the new model, shown in Fig. 2, the fit to the data was good (Chi-Square statistics 1.93, df = 5, *p* = 0.86, RMSEA = 0.000: and CFI = 0.99).

The revised model (Fig. 2) showed that depressive symptoms had a direct association to somatization (β = 0.83, *p* < 0.01), and a direct (β = 0.60, *p* < 0.01) as well as an indirect (β = 0.18, *p* < 0.01) association to cardiac anxiety. The total effect of depressive symptoms on cardiac anxiety was 0.78 (*p* < 0.01). By these paths, an indirect association between depressive symptoms and healthcare visits was found (β = 0.47, *p* < 0.01). Table 3 shows all indirect and total effects in the revised SEM model. Specific analysis of the model based on sex was tested, but no differences were found.

**Table 3:** Indirect and total effects in the revised structural equation model for the total sample of patients with non-cardiac chest pain, *N* = 552

	Depressive symptoms		Somatization		Fear of body sensations		Cardiac anxiety		Healthcare visits	
	Indirect	Total	Indirect	Total	Indirect	Total	Indirect	Total	Indirect	Total
Depressive symptoms	–	–	–	0.83	0.59	0.59	0.18	0.78	0.47	0.47
Somatization	–	–	–	–	–	0.71	0.21	0.21	0.13	0.13
Fear of body sensations	–	–	–	–	–	–	–	0.30	0.18	0.18
Cardiac anxiety	–	–	–	–	–	–	–	–	–	0.61

### The contribution of cardiac disease in the associations between psychological distress and healthcare use

**Fig. 3:**
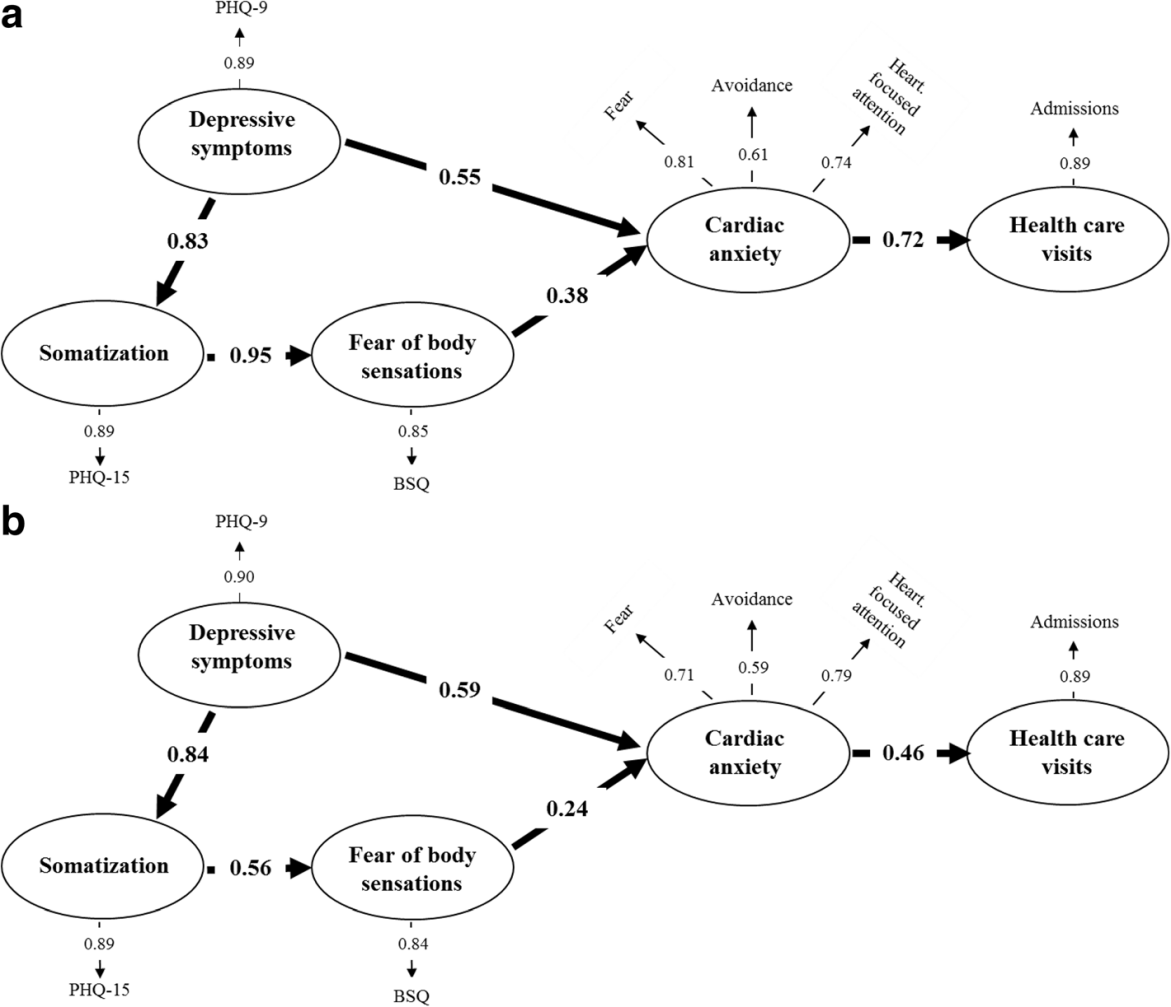
**a** + **b** The revised model of the associations between somatization, fear of body sensations, cardiac anxiety, depressive symptoms, and healthcare use in patients with non-cardiac chest with (**a** Chi-Square = 9.69, df = 10, *P*-value = 0.46772, RMSEA = 0.000, CFI = 0.99) and without history of cardiac disease (**b** Chi-Square = 4.25, df = 7, *P*-value = 0.75067, RMSEA = 0.000, CFI = 0.99). *BSQ* Body Sensations Questionnaire, *CAQ* Cardiac Anxiety Questionnaire, *PHQ-9* Patient Health Questionnaire-9, *PHQ-15* Patient Health Questionnaire-15

To explore the contribution of CD, two SEM models of the revised model were analysed; one including patients with history of CD (*n* = 188) and the other including patients without a history of CD (*n* = 360). Examination of the fit indices showed good fit of the model for both patients with a history of CD (Fig. 3a, Chi-Square statistics 9.69, df = 10, *p* = 0.47, RMSEA = 0.000, and CFI = 0.99) and for patients without (Fig. 3b, Chi-Square statistics 4.25, df = 7, *p* = 0.75, RMSEA = 0.000, and CFI = 0.99). All indirect and total effects in the two models are shown in Table 4. The differences in the effects between the groups were analysed using Fisher r to z transformation.

**Table 4:** Indirect and total effects in the revised structural equation model for non-cardiac chest pain patients with or without history of cardiac disease

	Depressive symptoms		Somatization		Fear of body sensations		Cardiac anxiety		Healthcare visits	
	Indirect	Total	Indirect	Total	Indirect	Total	Indirect	Total	Indirect	Total
Patients with history of cardiac disease, *n* = 188										
Depressive symptoms	–	–	–	0.83	0.79	0.79	0.30	0.85	0.62	0.62
Somatization	–	–	–	–	–	0.95	0.36	0.36	0.26	0.26
Fear of body sensations	–	–	–	–	–	–	–	0.38	0.28	0.28
Cardiac anxiety	–	–	–	–	–	–	–	–	–	0.72
Patients with no history of cardiac disease, *n* = 360										
Depressive symptoms	–	–	–	0.84	0.47	0.47	0.11	0.70	0.33	0.33
Somatization	–	–	–	–	–	0.56	0.13	0.13	0.06	0.06
Fear of body sensations	–	–	–	–	–	–	–	0.24	0.11	0.11
Cardiac anxiety	–	–	–	–	–	–	–	–	–	0.46

In general, both direct as well as indirect effects were stronger in the model representing patients with history of CD than in the model based on patients without history of CD. In patients with a history of CD, depressive symptoms had a significantly stronger indirect effect on fear of body sensations (β = 0.79 vs. β = 0.47, *p* < 0.01), cardiac anxiety (β = 0.30 vs. β = 0.11, *p* < 0.05), and number of healthcare visits (β = 0.62 vs. β = 0.33, *p* < 0.01) than in patients without a history of CD. Further, in patients with a history of CD, somatization had a significantly stronger direct effect on fear of body sensations (β = 0.95 vs. β = 0.56, *p* < 0.01), as well as stronger indirect effect on cardiac anxiety (β = 0.36 vs. β = 0.13, *p* < 0.01), and number of healthcare visits (β = 0.26 vs. β = 0.06, *p* < 0.01) compared to those without. Additionally, also fear of body sensations had a significantly stronger direct effect on cardiac anxiety (β = 0.38 vs. β = 0.24, *p* < 0.05), as well as stronger indirect effect on number of healthcare visits (β = 0.28 vs. β = 0.11, *p* < 0.05) in those with CD than in those without. Finally, the direct effect of cardiac anxiety on healthcare visits was significantly stronger (β = 0.72 vs. β = 0.46, *p* < 0.01) in patients with CD than in those without. The direct effects of depressive symptoms on cardiac anxiety were about the same in both groups (β = 0.55 vs. β = 0.59).

**Fig. 4:**
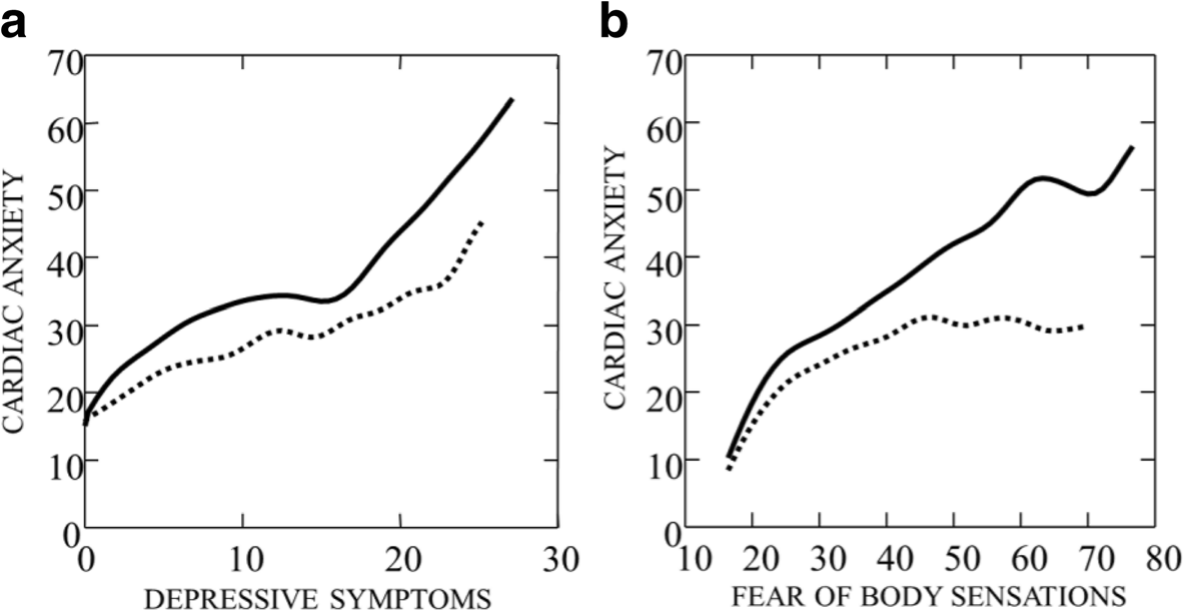
**a** + **b** Cardiac anxiety as a function of depressive symptoms and fear of body sensations (BSQ). Solid line indicates non-cardiac chest pain patients with history of, and dashed line chest pain patients with no history of cardiac disease. The lines are smoothed by means of distance-weighted least squares regression (DWLS)

Depressive symptoms and fear of body sensations seem to be important preconditioning factors for cardiac anxiety in patients with a history of CD. Figure 4a shows that higher scores on the depressive symptoms scale (> 15) for patients with a history of CD (solid line), are associated with cardiac anxiety to a substantially larger extent compared to in patients with no history of CD (dashed line). Figure 4b shows that body sensations are associated with cardiac anxiety all over the scale for patients with a history of CD (solid line), but for patients with no history of CD (dashed line) body sensations at scores higher than about 40 cease to induce increases in cardiac anxiety.

## Discussion

Our SEM analyses revealed that depressive symptoms were an important underlying variable, (i.e. lying behind other mechanisms) and influences healthcare use differently in patients with NCCP with or without history of CD.

There was no direct association between depressive symptoms and healthcare use, which suggests that depressive symptoms may act more as an underlying variable than an outcome as in the fear avoidance model [15], which is shown in our revised SEM model (Fig. 2). Our findings are supported by a 2015 study [30] using SEM analysis and showing that depressive symptoms had substantial impact on fear avoidance beliefs and physical functioning. In our revised model, depressive symptoms had strong direct effects on both somatization and cardiac anxiety, and indirect effects on fear of body sensation as well as cardiac anxiety. By these paths, depressive symptoms had a strong indirect effect on healthcare use. In addition, somatization had a strong direct effect on fear of body sensations and an indirect effect on cardiac anxiety. Therefore, in our model depressive symptoms and somatization can be seen as underlying variables. On the other hand, cardiac anxiety which is highly prevalent in patients with NCCP, can be seen as a state variable (i.e. temporarily induced by situations perceived as dangerous), and thus becomes the signal that drives patients to seek care in case of chest pain [13, 31]. In this case, cardiac anxiety is a result of an arising unfamiliar situation characterized by chest pain that is interpreted as threatening and driving the patients to contact healthcare providers.

The revised models based on history of CD acted relatively similar for both groups (Fig. 3a vs. b). Depressive symptoms had in both groups an indirect effect on healthcare visits, although this effect was significantly stronger in patients with history of CD than in those without. Furthermore, this effect was mediated by somatization, fear of body sensations, and cardiac anxiety. Additionally, all the direct and indirect effects between the different variables of psychological distress and healthcare use were significantly stronger in patients with history of CD.

In patients with history of CD, cardiac anxiety was substantially more associated with higher scores of depressive symptoms compared to those without history of CD (Fig. 4a). This can be explained by stronger associations between somatization and fear of body sensations, and also between fear of body sensations and cardiac anxiety. From Fig. 4b a linear relationship between cardiac anxiety and fear of body sensations was found for patients with history of CD, and a curved, leveling of relationship for patients with no history of CD. This means that, for patients without history of CD, there is an increase in cardiac anxiety as a function of fear of body sensations up to a certain level (about a scale value of 40), but above this level, increase in fear of body sensations has no effect on cardiac anxiety. Furthermore, the relationship between fear of body sensations and somatization shows the same difference as that found for the relationship between cardiac anxiety and fear of body sensations in Fig. 4b. Accordingly, in contrast to patients without a history of CD, higher levels of somatization were associated with higher levels of fear of body sensations, and higher levels in fear of body sensations were in turn associated with higher levels of cardiac anxiety in patients with history of CD. The difference between the groups in our study can be explained by the fact that patients with history of CD suffer from fear and anxiety due to earlier experience of physical symptoms [32–36]. In addition, one of the main components in cardiac rehabilitation is to teach patients to pay attention to and seek immediate care in case of persistent chest pain [20, 21]. This is in line with previous findings revealing that patients perceiving pain as threatening experience pain-related fear and safety seeking behaviour, such as avoidance [15, 37] and frequent visits to healthcare providers [38].

### Limitations

The response rate of about 30% was quite low, but not unusual when approaching a general population and is comparable with data from different surveys [39]. The study team had no access to medical charts and some of the patients may represent chest pain under evaluation, although their discharge diagnosis was NCCP. The diagnosis of CD was based on diagnosis of angina pectoris, myocardial infarction, and/or heart failure and can be seen as a limitation. Another limitation is that the causal relationship cannot be determined due to use of a cross-sectional design. In addition, the use of self-report data is a limitation. Although patient report has been found fairly accurate compared to physician report [22], this could be a limitation.

Further, patients with history of CD were older, had more comorbidities, larger number of healthcare visits, and reported significantly higher scores in all variables but fear of body sensations. This may mirror that it was not only the CD in itself that may explain the differences between the groups.

## Conclusions

Depressive symptoms only had an indirect association with healthcare use in patients with NCCP regardless of history of CD, and this association was mainly mediated by somatization, fear of body sensations, and cardiac anxiety. Hence, these psychological mechanisms play an important role in healthcare seeking behaviour, especially in those with history of CD, and lead to greater suffering for the individuals and higher healthcare costs. Therefore, there is a need for new innovative interventions targeting this patient group. Internet-delivered cognitive behavior therapy has been found feasible in this patient group. Although differences between the groups may be related to previous experience of a cardiac event, differences regarding group characteristics mentioned in the limitations could potentially confound the results. Therefore, more research is needed to clarify if such interventions should be tailored with regard to history of CD since this may have an impact on the way patients perceive, interpret, and act on their symptoms.

## Abbreviations

BSQ: Body Sensations Questionnaire
CAQ: Cardiac Anxiety Questionnaire
CD: Cardiac Disease
CFI: Comparative Fit Index
DWLS: Distance-Weighted Least Squares
ICD: International Classification of Diseases
NCCP: Non-Cardiac Chest Pain
PHQ-15: Patient Health Questionnaire-15
PHQ-9: Patient Health Questionnaire-9
RMSEA: Root Mean Square Error of Approximation
SEM: Structural Equation Modeling
